# Piloting a community-based psychosocial group intervention designed to reduce distress among conflict-affected adults in Colombia: a mixed-method study of remote, hybrid, and in-person modalities during the COVID-19 pandemic

**DOI:** 10.1186/s13033-023-00597-4

**Published:** 2023-10-24

**Authors:** Michel Rattner, Leah Emily James, Juan Fernando Botero, Hernando Chiari, Guillermo Andrés Bastidas Beltrán, Mateo Bernal, Juan Nicolás Cardona, Carlos Gantiva

**Affiliations:** 1 Department of Psychology, Palo Alto University, Palo Alto, CA 94304 USA; 2 Department of Psychology, Universidad de Los Andes, Bogotá, Colombia; 3 Heartland Alliance International, 208 S. LaSalle Street, Suite 1300, Chicago, IL 60604 USA

**Keywords:** Low-middle-income countries, COVID-19, Community-based, Group intervention, Lay-providers, Mixed methods, Remote, Hybrid, In-person

## Abstract

**Background:**

Community members in Quibdó (Choco, Colombia) are highly vulnerable to psychosocial problems associated with the internal armed conflict, poverty, and insufficient public services, and exacerbated by the COVID-19 pandemic. A pilot study was conducted with conflict-affected adults in Quibdó to assess feasibility and outcomes of a community-based psychosocial support group intervention using three different intervention modalities: in-person, remote (conducted online), and hybrid (half of sessions in-person, half-remote). This group model integrated problem-solving and culturally based expressive activities and was facilitated by local community members with supervision by mental health professionals.

**Methods:**

This study utilized a mixed-explanatory sequential design (a quantitative phase deriving in a qualitative phase) with 39 participants and 8 staff members. Participants completed quantitative interviews before and after an eight-week group intervention. A subset of 17 participants also completed in-depth qualitative interviews and a focus group discussion was conducted with staff at post-intervention.

**Results:**

From pre- to post-intervention, participants in all modalities demonstrated improved wellbeing and reduced symptoms of generalized distress, anxiety, depression, and posttraumatic stress. Use of coping skills varied across modalities, with remote groups associated with a decrease in some forms of coping, including use of social support. In qualitative interviews and the focus group discussion, participants and staff described logistical challenges and successes, as well as facilitators of change such as problem resolution, emotional regulation and social support with variations across modalities, such that remote groups provided fewer opportunities for social support and cohesion.

**Conclusions:**

Results offer preliminary evidence that this model can address psychosocial difficulties across the three modalities, while also identifying potential risks and challenges, therefore providing useful guidance for service delivery in conflict-affected settings during the COVID-19 pandemic and other challenging contexts. Implications of this study for subsequent implementation of a Randomized Control Trial (RCT) are discussed.

**Supplementary Information:**

The online version contains supplementary material available at 10.1186/s13033-023-00597-4.

## Introduction

Low- and middle-income countries (LMICs) represent approximately 85% of the global population and are characterized by unstable socio-political conditions, poverty, unemployment, low education, and violation of human rights, including exposure to conflict-related violence [1–3]. Such conditions are associated with an increased risk of mental health problems; over 70% of the psychopathological burden occurs in LMICs [4]. The COVID-19 pandemic has further heightened the vulnerability of these populations and exacerbated pre-existing social inequalities [5, 6].

Colombia is an LMIC with an internal armed conflict spanning over five decades. In 2022, the Colombian Victims Unit Registry [7] reported more than 11 million violent events and over 9 million victims, the majority of whom were internally displaced. Despite the signing of a peace agreement in 2016, Colombians continue to suffer from ongoing violence due to disputes between dissident illegal armed forces, narcoterrorism, and insufficient government enforcement of the agreement [8]. Widespread inequity (e.g., inhospitable living conditions, community violence, unemployment) continues to severely affect the country in both rural and urban areas [9]. Quibdó has a long history of exposure to armed conflict [10] and a high incidence of poverty (64.8%) compared to the national rate (39.3%) [11]. A minority of the population have access to adequate infrastructure (26.5%), sewage services (17.8%) and clean water (25.2%). In addition, Chocó has been one of the primary national receptors of internally displaced persons (IDPs) and Venezuelan refugees [11–13].

Research has shown high levels of posttraumatic stress disorder (PTSD), anxiety, and depression as well as impaired functioning in Colombian IDPs and other armed conflict victims [14–16]. The development of evidence-based interventions to support these populations is a priority in the mental health and psychosocial support (MHPSS) sector [12, 17]. However, LMICs often suffer from insufficient human and financial resources to provide MHPSS services to those requiring care, with estimates that close to 75% of those requiring care are unable to receive it [18, 19]. The World Health Organization [20] reported that Colombia suffers from an inequitable distribution of human health resources (including MHPSS), with Chocó being one of the departments with the greatest shortage (20.21 doctors/10,000 inhabitants) [21].

The COVID-19 pandemic has further exacerbated needs. Several international epidemiological studies have demonstrated deleterious effects of the COVID-19 pandemic and associated quarantine measures on populations worldwide (e.g., economic stress, job insecurity and unemployment, social isolation, decreased access to community support, educational and health services) and subsequent MHPSS consequences (e.g., anxiety, mood and trauma, and stress-related disorders, suicidal ideation and attempts) [22–24]. These effects are particularly pronounced in LMICs [19]. Colombia’s PSY-COVID study conducted with over 18,000 persons in 2020 revealed significant mental health outcomes associated with COVID-19 lockdown, such that 35% reported symptoms of depression and 29% symptoms of anxiety, with low-income, female, and young adults most at risk [25].

The WHO [26] has proposed that Task-Shifting Models (TSM), in which non-specialized health practitioners are trained and supervised to provide basic services traditionally performed by specialized personnel, can help to overcome resource shortages, and promote long-term capacity-building in LMICs. Moreover, this approach can promote cultural sensitivity and reduce mental health stigma, thus promoting acceptance, service uptake and adherence among the community members [17]. TSMs such as the Mental Health Gap Action Program (mhGAP), Problem Management Plus (PM+), Médecins Sans Frontières programs, and national mental health hotlines’ protocols, in which non-specialized personnel is trained to provide psychosocial support services, have helped to reduce the gap in MHPSS services availability in LMICs [6, 27–29]. This approach may be particularly critical during a pandemic, in which mental health needs are high and specialists are likely to be especially limited.

An additional strategy to meet needs during the COVID-19 pandemic is the adaptation of services to telemedicine via mobile health platforms, teleconsultation, and apps. These approaches aim to prevent the risk of infection during service delivery or transport to services, while also reducing costs and time [30]. The use of remote modalities may present particular advantages in LMIC contexts, such as addressing service delivery gaps through coverage in isolated or unsafe locations, ease of training and supervision, and circumvention of stigma. However, remote modality interventions have highlighted new challenges, especially among vulnerable populations who may not have access to connectivity, devices, or technological literacy. Difficulties also arise when adapting community-based psychosocial support (CB-PSS) group interventions to remote modalities which can create challenges for peer-to-peer interaction. Little research has examined the use of different modalities (i.e., remote, hybrid, in-person) for implementation of CB-PSS group interventions in LMICs and emergency settings [31].

### Current study

From 2010 to 2020, the Association of Organizations for Emotional Support (ACOPLE) program in Colombia provided MHPSS services to conflict-affected Afro-Colombians in Buenaventura and Quibdó using a TSM approach, such that services were facilitated by non-specialist community members (Community Psychosocial Agents, CPAs) with training and supervision by mental health professionals. The original model included implementation and testing of an individual intervention using Common-Elements Treatment Approach (CETA), which was shown to be effective in reducing symptoms of anxiety, PTSD and depression in Buenaventura, and PTSD symptoms in Quibdó [32]. It also included a CB-PSS group intervention drawing on a narrative therapy approach [17]. Research on this model highlighted the importance of including culturally based components grounded in the Pacific region’s traditional practices within the intervention protocols [17, 33, 34]. The group model evolved over time to remain responsive to participants’ needs, ultimately incorporating enhanced collective problem-solving components and culturally specific expressive elements into a final community-based group protocol to overcome cultural validity gaps [35]. When the pandemic began, raising concerns that in-person services would be unsafe or prevented by lockdown, the CB-PSS protocol was adapted to remote (conducted entirely by phone and internet) and hybrid (i.e., four problem-solving sessions delivered remotely, and the introductory plus three expressive sessions in-person) in addition to the in-person modality.

This mixed-method pilot study aims to provide preliminary evidence regarding the feasibility, outcomes, and acceptability of implementing a culturally adapted CB-PSS group intervention for armed conflict-affected adults from Quibdó, Colombia, delivered in three modalities (i.e., remote, hybrid, and in-person) by local lay providers, with focus on the following questions:

1. Is the intervention feasible?
  - What are the main facilitators and barriers to implementation?
2. Is participation in this intervention associated with improvement in key distress (PTSD, anxiety, depression, functional impairment), psychosocial wellbeing (wellbeing and community efficacy), and coping strategies outcomes?
3. Is the intervention acceptable to participants?
  - In particular, were participants responsive to cultural adaptation strategies?

Results are expected to strengthen the methodology for a subsequent randomized controlled trial (RCT) and to offer a unique and timely contribution regarding best practices in the provision of CB-PSS group interventions using a TSM approach delivered by trained local lay providers during the COVID-19 pandemic.

## Method

### Design

A mixed explanatory-sequential research method study was conducted with two main phases: (1) quantitative: pre-experimental pretest–posttest single group design and (2) qualitative: cross-sectional phenomenological interpretive design [36, 37]. Based on the two previous phases, an expansion/elaboration process was conducted to inform interpretations [38].

### Participants

The sample entailed adult (age 18 or over) participants residents of Quibdó who were affected by armed conflict-related violence were eligible to participate. Exclusion criteria were significant risk of suicide/self-harm or psychosis. These were screened using a pre-assessment quantitative questionnaire designed by HAI to identify and manage these cases. An initial sample of 60 participants gave informed consent and completed pre-assessment measures, then were randomly assigned to participate in remote, hybrid, or in-person modalities. However, 21 participants were excluded from analyses because their pre-assessments could not be completed prior to beginning the first session, were referred to specialized services (*n* = 7), or because they did not complete the post-test, resulting in a final sample of 39 participants. Sociodemographic distribution by modalities is presented in Table 1.

**Table 1:** Socio-demographic distribution by modality

Variables		Remote	In-person	Hybrid	By modality differences, significance level
		*(n* = *12)*	*(n* = *13)*	*(n* = *14)*	*X*^*2*^* or F*
Gender *n* (%)	Men	7 (41.6)	0 (0)	4 (28.6)	6.74*
	Women	5 (58.4)	13 (100)	10 (71.4)	
Age (years)	Range	18–28	19–47	18–29	
	M (SD)	21.28 (3.23)	29.23 (10.02)	22.79 (3.49)	5.28*
Ethnicity *n* (%)	Afro-Colombian	12 (100)	13 (100)	14 (100)	
Education level *n* (%)	Primary school or less	2 (16.7)	1 (7.7)	0 (0)	9.45
	Middle to high school	8 (66.7)	3 (23.1)	5 (35.7)	
	Undergraduate degree or higher	2 (16.7)	9 (69.2)	9 (64.3)	
Marital status *n* (%)	Single	10 (83.3)	9 (69.2)	78.6	
	Married/in a relationship	2 (16.7)	3 (23.1)	21.4	2.30
	Divorced	0	1 (7.7)	0	
Employment status *n* (%)	Employed	6 (50)	2 (15.4)	4 (28.6)	3.65
	Student	3 (25)	6 (46.2)	5 (36.7)	
	Housework	2 (16.7)	3 (23.1)	3 (21.4)	
	Unemployed	1 (8.3)	2 (15.4)	2 (14.3)	
Internally displaced *n* (%)	Yes	7 (58.4)	7 (53.8)	11 (78.6)	6.41*
	No	5 (41.6)	5 (38.4)	0	
	Missing data	0 (0)	1 (7.7)	3 (21.4)	
Traumatic events (7 max)	Range *M (SD)*	0–7 2.67 (1.41)	0–7 2.25 (1.96)	0–7 3.10 (2.03)	0.44

*Significant group differences at* p* < 0.05

Twenty-four participants who completed the quantitative phase were invited to participate in individual in-depth, semi-structured qualitative interviews. These participants were randomly selected from the full sample while ensuring representativeness across modalities. Seventeen (female *n* = 12, male *n* = 5; remote *n* = 5, in-person *n* = 6, hybrid *n* = 6) participated, with the remaining declining or dropping out due to conflict with daily activities, failure to answer or disconnected phones.

**Fig. 1:**
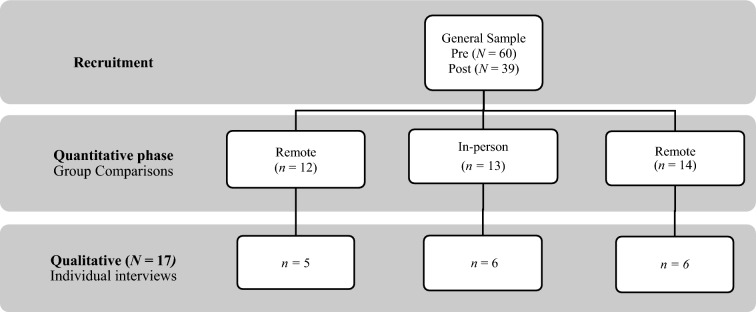
Pilot study phases

Finally, all eight staff members were invited to participate in a focus group discussion (FGD) held after the intervention ended. Six staff members attended, consisting of four CPAs and two professionals (1 psychologist and 1 social worker) (Fig. 1).

### Instruments

#### Quantitative phase

The pre-assessment quantitative questionnaire entailed three sections: sociodemographic factors (see Table 1), primary outcomes (see Table 2), risk screening semi-structured interview and risk protocol (i.e., suicide, general violence exposure, and perpetration of violence), a 3-item section for the interviewer to rating potential psychotic symptoms, and an additional seven items dichotomous (Yes/No) of direct and witnessing potentially traumatic experiences scale (e.g., *At any point in your life has anyone assaulted, sexually abused or raped you?*). The post-assessment questionnaire included the primary outcome measures and a final 16-item section assessing participants’ satisfaction with the intervention in which participants were asked to indicate agreement with 14 items (e.g., *To what extent did you feel supported in the group?*).

**Table 2:** Primary outcome measures

Variable	Scale	Instrument	Description	Cronbach α
Wellbeing	0–10	Personal Wellbeing Index (39)	Seven items assessing perceived quality of life (life, health, relationships, security, community connection, future security)	0.72
Generalized distress	1–5	Kessler-6 [40]	Six items assessing generalized psychological distress	0.55
Depression	0–3	Hopkins Symptoms Checklist -HSCL-25 [41]	25 items, 15 for depression symptoms and 10 for anxiety symptoms	0.89
Anxiety	0–3			
PTSD	0–3	PTSD Checklist Civilian-PCL-C [42]	16 items assessing symptoms of posttraumatic stress disorder (PTSD) in a civilian population	0.89
Functional impairment	1–5	WHO Disability Assessment Schedule WHO-DAS [43]	12 items assessing impaired ability to function across six life domains (household, cognitive, mobility, self-care, social, society)	0.83
Community efficacy	1–5	Community efficacy scale [44]	Eight items assessing community efficacy, defined as group capability to perform actions towards a common goal	0.81
Coping Strategies	1–4	Brief Cope [45, 46]	2-item-14 subscales (active coping, planning, positive reframing, acceptance, religion, humor, emotional support, social/instrumental support, behavioral disengagement, venting, self-distraction, denial^a^, self-blame, and substance use) assessing the use of coping strategies	0.86

^a^The denial subscale was removed from the study due to an error in the platform on which the instruments were uploaded and administered

#### Qualitative phase

Categories for the qualitative interviews and the descriptive analyses (Lv1) are presented in Table 3. Operational definitions were proposed and validated for the categories, based on WHO’s [26, 47] ecological model and Leavy’s [38] phenomenological considerations**.**

**Table 3:** Themes and subthemes

Descriptive subthemes (Lv1)	
Commitment	Factors contributing to participant engagement
Feasibility	Factors impeding and encouraging participant attendance
Adaptability	Cultural fit of the intervention
Utility	Effectiveness of the intervention in addressing psychosocial needs
Scalability	Potential conditions for adapting and scaling-up the intervention to other populations
Satisfaction	Overall level of satisfaction
Adoption (only for staff)	Willingness to adopt the model of intervention

Based on relations of descriptive subthemes (Lv1) analytic subthemes (Lv2) emerge- contained into analytic themes (LV3)			
Themes (Lv3)	Description	Subthemes (Lv2)	Description
Contextual factors and strategies to benefit feasibility	Biopsychosocial challenges and resources stemming from the context and strategies to address challenges and capitalize on resources	Community challenges	Contextual challenges for the implementation of programs in Quibdó
		Community resources	Contextual resources that facilitate the implementation of programs in Quibdó
		Engagement and retention strategies	Implemented strategies by this program to overcome challenges and potentiate resources
Intervention outcomes and acceptability	Participant outcomes, perceived facilitators of change, and acceptability/satisfaction associated with the intervention	Outcomes and facilitators of change	Intervention benefits and facilitators for improving participants’ skills and wellbeing
		Acceptability	Participants’ satisfaction with the program
		Cultural Fit	Cultural appropriateness of the program for participant comprehension and adjustment to their culture and costumes
		Staff Satisfaction	Staff perception of the program including logistics, training, supervision, and implementation

The qualitative protocol utilized two instruments based upon the Lv1 descriptive subthemes: (1) an individual semi-structured interview schedule made up of 23 open-ended questions intended to explore the participants’ experiences in the intervention and (2) an FGD protocol with eight questions designed to explore the experience of the staff while implementing the intervention.

### Procedure

The CB-PSS group intervention was conducted between October and December 2020, when lockdown measures were uplifted, but biosecurity alerts remained highly active. Participants were recruited using a non-probabilistic snowball sampling approach, drawing from CPA’s networks of local organizations, leaders, and the Quibdó Mayor’s office. Information meetings with community councils and neighborhood associations’ leaders, resulted in leaders inviting participants to voluntarily receive an individual informational meeting, where they decided if signing-up. Recruitment also included advertisements via social media (i.e., WhatsApp and Facebook) and posters.

The groups lasted over eight weekly sessions of approximately 2 h. The intervention consisted of an introductory session, three sessions focused on collaborative problem-solving drawing from the Problem Management Plus protocol [48], and four sessions focused on expressive activities based on cultural practices and designed to benefit emotional regulation summarized in Appendix [35]. Each group was facilitated by two CPAs and was supervised by a psychologist or social worker who also provided training, evaluation, and weekly supervision sessions to all the CPA’s team. Moreover, the psychologist and social worker had weekly supervision by the mental health manager to ensure fidelity, adjustment, and safety when implementing [33]. All staff members had previous experience conducting CB-PSS services with the ACOPLE project in Quibdó. Three groups in each modality (i.e., nine groups total), each with an average of seven participants across the eight sessions.

As mentioned, due to the contextual challenges imposed by the COVID-19 pandemic, the intervention was conducted in three modalities to increase accessibility and safety. The in-person sessions were conducted face-to-face in safe and accessible community locations (e.g., community center, school) with bio-security measures (e.g., social distancing, masks, hand gel), while the remote sessions were conducted via the Zoom platform using audio, video, and chat with an option to call in by phone as needed. Hybrid groups were compound by both types of sessions as mentioned above. Prior to implementation, the team received training and shared input to adapt intervention contents to each modality and to address logistical considerations.

#### Data collection, management, and analysis

*Quantitative Phase* The pre and post-intervention quantitative questionnaire data were collected by the CPAs using the KOBO Toolbox platform on tablets. Data were cleaned and systematized in a Microsoft Excel database (see Additional file 1: Pilot Dataset). Descriptive and inferential analyses were performed using SPSS; mixed analyses of variance (ANOVA) of repeated measures were run for the measures of interest. Post hoc multiple comparison analyses adjusted with Bonferroni correction were performed. Person mean substitution method was used to address missing data. The level of significance was set at *p* < 0.05. Finally, to assess evaluator bias, the long string index of responses averaged by CPAs at pre and post was examined.

*Qualitative Phase* One week after completing post-intervention questionnaires, the qualitative protocol was conducted by trained psychologists, and recorded in audio software on password-protected smartphones, then downloaded to secure computers. The recordings were transcribed on Microsoft Word document by trained undergraduate psychology students. Afterward, the transcripts were divided by respondents (participants, staff) and by participant modality (i.e., in-person, remote, hybrid) and were randomly assigned to four research team members to minimize interpretation bias. Each researcher processed the data in a qualitative Excel matrix and NVivo software. Lastly, phenomenological thematic links and interpretations by participant modalities and staff were based upon researchers' comparisons and agreements deriving the analytic subthemes (Lv2) and themes (Lv3) definition (Table 3).

### Ethical considerations

Ethical approval was received from Heartland Alliance International (IRB00004277), and Universidad de Los Andes (Acta No 1305 de 2021) institutional review boards (IRBs) prior to commencing the study. All procedures were conducted in accordance with the declarations of Helsinki. Individual consent processes were conducted with each participant, including providing hard copy consent forms which were read aloud to participants. A single consent form was used for the quantitative interviews (pre and post) and the intervention, and additional separate consent forms used for the qualitative participant interviews and staff FGD. Participants provided verbal informed consent, which was recorded by interviewers (no participant names were recorded). All participant data was identified using codes (e.g., H0001), and stored in secure research team computers. All in-person activities included biosecurity measures described in the procedure.

## Results

### Quantitative phase

#### Wellbeing and distress outcomes

Mixed repeated measures analyses of variance (RM-ANOVA) were conducted for the variables of interest, with Modality (i.e., in-person, remote, and hybrid) as between-subject factor and Time (i.e., pre and post) as within-subject factor (Table 4). A significant main effect of Time was found, such that participants demonstrated a significant increase at post-intervention in wellbeing (*F*_(1,36)_ = 30.07, *p* < 0.001, η_p_^2^ = 0.45) and decreased scores at post-intervention for generalized distress, (*F*_(1, 36)_ = 37.95, *p* < 0.001, η_p_^2^ = 0.51), and symptoms of depression (*F*_(1,36)_ = 40.83, *p* < 0.001, η_p_^2^ = 0.53), anxiety (*F*_(1,36)_ = 49.33, *p* < 0.001, η_p_^2^ = 0.57) and PTSD (*F*_(1,36)_ = 41.10, *p* < 0.001, η_p_^*2*^ = 0.53). No significant differences were found between pre and post-intervention in functional impairment (*p* = 0.09) or community efficacy (*p* = 0.74). No significant main effects by Modality or Modality × Time interactions were found for any of these variables (all *p* > 0.05).

**Table 4:** Wellbeing and distress outcomes

Variables	Pre-test *M (SD)*	Post-test *M (SD)*	*F*
Wellbeing	41.82 (10.61)	56.13 (13.01)	30.07***
Generalized distress	17.53 (3.77)	12.08 (5.12)	37.95***
Depression	23.36 (9.32)	8.76 (8.96)	40.83***
Anxiety	14.18 (7.10)	4.08 (4.85)	49.33***
PTSD	25.56 (11.23)	7.90 (9.43)	41.10***
Functional impairment	20 (7.35)	16.97 (7.09)	2.99
Community efficacy	27.62 (6.92)	27.97 (5.50)	0.10

***p < 0.001

#### Coping strategies by modality

A significant effect of Time was found for the religion subscale (*F*_(1,36)_ = 5.46, *p* = 0.02, η_p_^2^ = 0.13), such that scores increased at post-intervention. The ANOVA revealed a significant main effect of Modality for coping subscales of social/instrumental support (*F*_(2,36)_ = 8.45, *p* = 0.001, η_p_^2^ = 0.32), religion (*F*_(2,36)_ = 5.75, *p* = 0.007, η_p_^2^ = 0.24), and self-distraction (*F*_(2,36)_ = 4.94, *p* = 0.01, η_p_^2^ = 0.21). Participants in in-person and hybrid modalities had higher scores in social support and religion subscales in comparison with remote modality (all *p* < 0.04). Those in the hybrid modality reported higher scores in self-distraction than in-person and remote modalities (both *p* < 0.05).

Significant Modality × Time interactions were found for coping subscales of emotional support (*F*_(2,36)_ = 4.19, *p* = 0.02, η_p_^2^ = 0.18), social/instrumental support (*F*_(2,36)_ = 6.03, *p* = 0.005, η_p_^2^ = 0.25), positive reappraisal (*F*_(2,36)_ = 4.14, *p* = 0.02, η_p_^2^ = 0.18), acceptance (*F*_(2,36)_ = 4.49, *p* = 0.01, η_p_^2^ = 0.20), and venting (*F*_(2,36)_ = 5.09, *p* = 0.01, η_p_^2^ = 0.22). Participants in the remote modality showed decreased emotional support (*p* = 0.04, *d* = 0.89), social support (*p* = 0.01, *d* = 0.86), positive reappraisal (*p* = 0.01, *d* = 0.73) and venting (*p* = 0.04, *d* = 1.04). Those in the Hybrid modality demonstrated increased acceptance (*p* = 0.004, *d* = 0.62) and venting (*p* = 0.03, *d* = 0.60). Finally, those in the in-person modality demonstrated increased emotional support (*p* = 0.05, *d* = 0.89) and social/instrumental support (*p* = 0.02, *d* = 0.91) scores at post-intervention (Table 5).

**Table 5:** Brief-COPE subscales, time × modality interactions

Variables	In-person		Hybrid		Remote		*F*
	Pretest *M (SD)*	Posttest *M (SD*)	Pretest *M (SD)*	Posttest *M (SD)*	Pretest *M (SD)*	Posttest *M (SD)*	
Emotional support	2.50 (0.73)	3.19 (0.77)	3.10 (1.00)	3.21 (1.12)	3.12 (0.90)	2.37 (0.77)	4.19*
Social/instrumental Support	2.84 (1.04)	3.57 (0.70)	3.50 (0.67)	3.57 (0.67)	3.04 (0.91)	2.16 (0.86)	6.03*
Positive Reappraisal	2.46 (0.77)	2.80 (0.80)	3.07 (1.03)	3.39 (0.90)	3.08 (0.99)	2.25 (0.78)	4.14*
Acceptance	2.69 (0.75)	2.92 (0.67)	2.89 (1.12)	3.57 (0.91)	2.70 (0.96)	2.41 (0.76)	4.49**
Venting	2.34 (1.02)	2.73 (0.94)	2.50 (0.65)	3.17 (0.93)	2.75 (0.58)	2.04 (0.62)	5.09**

*p < 0.05, **p < 0.01

#### Acceptability/satisfaction measures

At post-intervention, participants reported high satisfaction with the intervention overall (Table 6). An ANOVA yielded significant differences in feeling listened to (*F*_(2,37)_ = 3.60, *p* = 0.03, η_p_^2^ = 0.17) across modalities, such that those in the remote modality reported lower levels of feeling listened to than those in the in-person (*p* = 0.03) and hybrid (*p* = 0.03) modalities.

**Table 6:** Acceptability measures by modality

Variables	Remote *M (SD)*	In-person *M (SD)*	Hybrid *M (SD)*	*F*
Accessibility of groups	3.17 (1.26)	3.73 (0.90)	3.00 (1.35)	1.16
Satisfaction with group duration	2.50 (0.67)	2.73 (0.46)	2.77 (0.43)	0.88
Felt supported	3.75 (0.62)	3.91 (0.30)	3.69 (0.75)	0.40
Felt listened to	3.58 (0.79)	4.00 (0.00)	4.00 (0.00)	3.31*
Felt understood	3.33 (0.88)	3.73 (0.46)	3.69 (0.75)	1.06
Group was adapted to the culture	3.67 (0.77)	4.00 (0.00)	3.92 (0.27)	1.55
Willing to express self emotionally	3.58 (0.66)	3.82 (0.60)	3.77 (0.59)	0.46
Felt confidentiality protected	3.92 (0.28)	3.73 (0.64)	3.92 (0.27)	0.77
Learned new skills/tools	3.58 (0.99)	3.91 (0.30)	3.85 (0.55)	0.74
Overall satisfaction	3.67 (0.77)	4.00 (0.00)	4.00 (0.00)	2.20

*p < 0.05

Response scale 1 to 4

### Qualitative phase

As a result of the phenomenological-interpretative analyses (Table 3), the main findings were synthesized within the analytic themes and their correspondent analytic subthemes.

#### Contextual challenges, resources, and strategies to address

*Community challenges* Across all modalities, respondents reported challenges faced at the community level, often concerning lack of financial resources, unpredictability of daily activities, and the impact of COVID-19. Many participants reported insufficient access to meals, transportation, and technological devices/data. Moreover, many shared negative perceptions of mental health services in the community, due both to poor accessibility and quality (e.g., long delays in scheduling appointments) and to stigma (e.g., services are “for the crazy ones”). Secondly, a high percentage of community members reported engaging in informal work with a variable schedule and described the need to prioritize work (and associated fulfillment of basic needs) over other activities, including MHPSS. The COVID-19 pandemic exacerbated all these challenges; across modalities, participants reported that many community members lost their jobs and businesses and turned to alternative sources of income, including informal labor, and in one case reported a family member engaging with a gang to provide an income. Likewise, taking on additional responsibilities due to the lockdowns (e.g., additional childcare, including supervising children’s remote schooling) on top of their usual tasks (e.g., work, study, household tasks) was common. Thus, many participants struggled to participate regularly in CB-PSS groups.

Further, each session modality (i.e., remote and in-person) presented its own challenges. Participants in remote sessions described restricted access to technological devices and data, and connectivity problems (i.e., heavy rain dampening mobile and phone signal), and difficulties ensuring privacy and confidentiality during calls (i.e., family members, customers, colleagues overhearing or interrupting). A participant explained challenges regarding safety and confidentiality in remote sessions as follows:

*…folks are very messy and get out from the “seños” [CPA] control. And everybody speaks and there is the brother screaming… so you don’t feel with confidence to talk. Some of our topics have to be with family violence. Thus, we could say for example if they are interviewing you and ask you about this, and you are willing to tell what’s happening. But for me hearing the brother or anyone else, I don’t feel confident. Therefore, it is better in-person* (Hybrid group participant).

For the in-person sessions, participants shared concerns about lacking the necessary economic resources for ensuring food and transportation, worries about the possibility of attending sessions with people perceived as a risk, and security concerns such as crossing invisible territorial boundaries (i.e., bans imposed by illegal armed forces to prevent access to those from rival neighborhoods) to attend groups. Regarding in-person invisible territorial boundaries one participant explained: *“…[CPAs]…need to find a place to do the workshops because some folks don’t like to get around there. Because is a neighborhood where you can’t enter… you can’t pass by…I had a friend that only attended once, he said he didn’t like going around there”* (Hybrid group participant).

*Community resources* Despite these challenges, participants and staff shared important *community resources* that improved engagement and retention in MHPSS programs. Staff shared that the knowledge, credibility, and influence of well-accepted local actors (i.e., official institutions, community leaders and NGOs), are key for building trust and contacting the community, providing critical information (i.e., logistics, security), and supporting the program’s activities (e.g., encouraging buy-in, recruitment and retention support). In addition, participants remarked that the CPAs’ expertise both in MHPSS concepts and in understanding local community characteristics (drawing from their experiences as members of the same communities) were also critical.

An additional community resource was the use of traditional coping mechanisms to overcome difficult situations. Respondents shared that traditional coping practices (e.g., use of herbs, consultation with spiritual leaders, community rituals, folkloric and traditional practices) help to decrease distress and increase social bonding in the community: *"Let’s say, here in Chocó, each person has their way of managing their painful situations. Some believe, let’s say, in their religions, others in their culture, others in their ancestors, and so on"* (Hybrid group participant).

*Engagement and retention strategies* Respondents described engagement and retention strategies used by the project to overcome community-level challenges and build on community resources. Participants mentioned that validation of community leaders and the trajectory of ACOPLE for accepting the individual information meetings were important factors of engagement as one of them endorsed:

*I found out because I knew already ACOPLE, but I didn’t know how they worked…one time, they came to do a meeting, so a neighbor invited me and there they asked me if I would accept to participate in the group and that’s how I commenced to know them because I’ve already read ACOPLE’s poster, but I didn’t know the goal of the group* (Hybrid group participant).

Respondents highlighted that another value of collaborating with local community leaders was their intermediating role for provision of information, facilitation of contact, and follow-up with participants and CPAs throughout the program.

Both participants and staff also mentioned the importance of safety and security measures to ensure both engagement and retention. For the in-person sessions, most participants reported feeling safe in the designated locations to conduct the group, with few exceptions due to territorial barriers and the bio-safety measures (e.g., PPEs and protocols) to prevent COVID-19 infection. For the remote sessions, a safety-planning guideline was developed and shared with participants before groups began to guide participants in ensuring safe and confidential conditions in the household.

Across modalities, flexible scheduling options were presented and collectively agreed upon prior to beginning. Additional useful strategies included the provision of resources, including transport and mobile data stipends, a lending library for smartphones, biosafety materials, and snacks. Moreover, most participants stated that the CPA’s empathetic relationship style and follow-up measures (e.g., informal catch-up conversations/calls, and summaries at the beginning of sessions to reengage participants that missed the previous one) were important elements for ensuring engagement and retention.

*I think [CPAs are] very good because they were very caring, very on top of the things that you did, and it was common for people to call you, write you, ask you why you didn’t participate, why this, why that, and they always sent you information to do the session despite not participating. They gave you the guidelines on how to do the activities *(Remote group participant).

Participants with more complex needs than could be met by the group received referrals to the National Health System for specialized MHPSS services. Both participants and staff observed that these referrals were highly ineffective (i.e., appointments unavailable, insufficient number and duration of sessions, the excessive wait time before and between consultations, and poor-quality services available). No alternative referral options were reported by staff or participants.

#### Intervention outcomes and acceptability

*Outcomes and potential facilitators of change* Participants reported benefits from the CB-PSS groups associated with social support (cohesion, group bonding through activities, and group support to translate skills to daily life), peer learning (learning from others’ experiences and examples to display in daily life), emotional regulation, and problem-solving skills. Participants in in-person and hybrid groups emphasized the importance of the *collaborative style* of the group which allowed both for social support and peer learning to better manage problems and emotions in their daily lives. Hybrid participants shared that they preferred the in-person sessions to the remote sessions because they permitted more intimate contact without interruptions.

*One of the sessions that motivated me the most was to learn about situations…within the community environment, you're seeing the neighbor every day, right? But you don't really know, really what it is that torments the neighbor? What does the neighbor like? What doesn’t she like? What is it that makes her be quiet?* (In-person group participant).

In contrast, remote group participants described an *instructional style* into their group, similar to “taking classes”, with a focus on receiving information from the facilitators, participating, and hearing from other participants, but with less emphasis on the interactions and bonding with other members. However, remote group participants highlighted that this was preferred by some participants, such as those who desired flexibility to do other activities and those who did not feel comfortable with an active participatory style. Remote participants generally expressed appreciation for the content shared by the CPAs (with less emphasis on social support elements), as shared by one participant:

*So, I asked the psychologists in there, why this or that… I’ve read a lot about anxiety and being in courses, but to know how to really handle these things, meditate, and do the exercises… I’ve learned a lot of things in there* [The group]*, breathing techniques… and I’ve asked all that to them, and them the ones from ACOPLE explained to me […] they gave me an answer that made me feel satisfied… It was big because I’ve said excellent, I’m doing the things right […] what I did not understand I’ve asked, and they explained three, four times* (Remote group participant).

CPAs also described difficulties managing participants’ emotional reactions remotely due to participants more easily becoming disengaged and, in some cases, voluntarily disconnecting, especially during emotionally intense moments.

### Acceptability

Interesting differences arose across the intervention modalities regarding acceptability and satisfaction. Participants shared remote sessions allowed them to avoid the risk of community violence encountered when traveling to community centers, allowed for ease of scheduling amongst daily activities and were generally preferred by technology-savvy young people. However, respondents also reported frequent connectivity problems (sometimes due to bad weather) and privacy challenges related to household members overhearing or interrupting sessions remained even though the project strategies were implemented. Both staff and participants reported that competing activities (e.g., participants working or taking care of children during sessions) led to frequent interruptions.

*...as I told you about work, sometimes I have to stand up, or I can’t listen, and I had to go to take care of the clients and is very uncomfortable. I suppose to pay attention to a class you must be in an adequate place and be focused only on what is being done, and I had to do both things at the same time. Pay attention to clients and listen* (Remote group participant).

In contrast, the accessibility, social support, group cohesion and security of the spaces used during in-person sessions were highly valued in both in-person and hybrid modalities. The main difficulties for in-person sessions were attendance difficulties due to competing daily activities.

*Cultural fit* When asked about *cultural fit*, participants in all modalities emphasized that the empathetic relational style of the staff and that their status as local community members allowed them to better understand participant needs and to make group content more engaging and understandable. From the staff's perspective, the intervention protocol lacked specificity regarding cultural adaptation. However, they explained that the protocol was flexible enough to make spontaneous adaptations based on their own expertise as community members and the input from participants. CPAs created safe, judgement-free spaces for participants to direct sessions by sharing their own values and mechanisms for handling difficult situations, often stepping in to reinforce participants’ expertise. Participants recognized and appreciated the CPAs relational style and expertise:

*I didn’t see what to improve. There was empathy, good articulation. For me, well we were the ones to set up the conditions. It’s not something they came to impose. Thus, I think everything was done in a clear way, I mean each topic was visualized and worked. There was that great articulation among all: [the facilitation] team and the community members. Everything was beautiful* (In-person group participant)

Both staff and participants remarked on the importance of considering the diversity of the different cultural backgrounds across participants as a member of the groups remarked: *“Here there are different cultures and there was respect for each one’s beliefs. Here, we the afros are more… more into the body, while the indigenous are more into the rituals… Thus, each one must be considered […].”* (Hybrid group participant).

*Staff satisfaction* Across all modalities, staff members shared that they highly valued expressions of gratitude from participants and that this motivated their daily work. They also reported that they benefited personally from facilitating the CB-PSS groups in that participants’ contributions helped them to cope with personal situations. However, both participants and staff uniformly expressed frustration regarding difficulties with complex mental health needs and accessing specialized MHPSS referrals. As a staff member stated: *"Satisfied because, say, we achieved what was stipulated in the protocol regarding the sessions, right? But there were other participants’ needs that, I don’t know, weren’t met because there wasn’t an opportunity"* (CPA).

Staff shared that capacity building in the form of training and technical supervision from the MHPSS clinical director increased their ability to implement the protocol with fidelity and cultural sensitivity. However, staff mentioned that they would have benefited from additional applied drills and discussions regarding ways to handle the particular contextual problems and needs of the participants in Quibdó, especially during COVID-19. They also noted the needs for additional specific training to deal with crises in the remote sessions (i.e., remote psychological first aid and safety planning guidelines). The staff emphasized that weekly team planning meetings, which included professional supervision and peer-led exchange of lessons learned, were critical for overcoming these gaps.

## Discussion

This pilot study aimed to explore the feasibility, outcomes, and acceptability of a culturally adapted CB-PSS. Important lessons for cultural validity [9, 21, 22] and implementation [12, 15–17, 19, 20] of interventions resulting from the study intend to explore these gaps in the literature regarding best practices in community-based psychosocial support services in LMICs during COVID-19 and inform the subsequent RCT study. The results of this pilot demonstrated preliminary support for the acceptance and feasibility of the model, improving outcomes among victims of the armed conflict in Quibdó. Overall, quantitative and qualitative data revealed benefits associated with intervention participation across in-person, hybrid, and remote modalities. These findings support the utility of task-shifting through training non-specialized local lay providers to deliver CB-PSS services, using culturally adapted methods, and employing a diversity of modalities to bolster the acceptability, feasibility, and effectiveness of CB-PSS during the COVID-19 pandemic [29, 49]. However, important general challenges such as the prevalence of young, females, who majorly experienced violence, and are under challenging financial circumstances coincides with the most vulnerable groups mentioned in the PSY-COVID study [25] in the Colombian context due to COVID-19 and pre-existent vulnerabilities. In detail, particularities in terms of acceptability, feasibility and outcomes across modalities are going to be expanded in the following sections.

### Feasibility and acceptability

Overall, intervention implementation was shown to be feasible in all three modalities. All participants expressed similar levels of satisfaction (i.e., except for participants in the remote modality feeling less “listened to” than those in the other modalities), and all conveyed feeling safe, including from COVID-19 infection. However, qualitative data revealed unique strengths and weaknesses in participant engagement and retention. For example, in-person participants appreciated the social support elements of the meetings but struggled with scheduling and transportation. In contrast, remote participants appreciated the flexible scheduling and ability to avoid public transportation and potential exposure to community violence but faced connectivity and privacy challenges. According to staff, participants in remote sessions were also harder to keep engaged and sometimes logged off during emotionally intense moments. Although hybrid participants acknowledged the benefits of both modalities, they highlighted the value of the in-person sessions over the remote ones. Of note, there were no men in the in-person modality and fewer men than women in the hybrid modality, while gender distribution in the remote modality was relatively equal. Participants in the remote and hybrid modalities were younger than those in the in-person modality. Results suggest that all three modalities may prove valuable for different populations and in different contexts with safeguards and supports in place. For example, technologically savvy participants with access to devices and connectivity and busy schedules may find remote participation more feasible, while those who value face-to-face contact and have the necessary schedule availability may be more likely to engage in and benefit from in-person groups. Hybrid groups may strike a helpful balance between the two approaches for some participants with mixed needs and conditions.

### Outcomes and potential facilitators of change

Although interpretability of results is limited by small by-modality sample sizes and lack of a comparison group, outcome data offers useful insights to guide future research. Quantitative data demonstrated participant improvement in wellbeing, generalized distress, depression, anxiety, and PTSD outcomes from pre- to post-intervention across all modalities (in-person, hybrid, and remote). Overall, the qualitative results suggested improved functioning and benefits associated with applying the coping and problem-solving strategies learned in the groups in all modalities. Nevertheless, results also revealed notable differences in *coping strategies* and *facilitators of change* between modalities.

Those in the in-person modality demonstrated significant improvement in the use of social support as a coping mechanism (measured using the Brief COPE) from pre- to post-intervention. Likewise, qualitative data suggested that in-person group participants derived particular benefits from social support and related factors such as social cohesion, peer-to-peer identification, validation, and social learning. These participants shared that being listened to and understood by others and sharing common experiences and problems led to a sense of group identity and trust, and the ability to learn from others—factors that represent necessary conditions to instill change in group interventions [50].

Although the hybrid group participants did not significantly improve from pre- to post-intervention in the use of social support as a coping strategy, this may be attributed to a ceiling effect because the baseline score (*M* = 3.50, *SD* = 0.67) was already quite high. Moreover, post-intervention social support in the hybrid group (*M* = 3.57, *SD* = 0.67) did not differ from that of in-person modality participants (*M* = 3.57, *SD* = 0.70) significantly. These findings concur with qualitative data showing that hybrid group participants described benefitting from social support, including sharing experiences and exchanging support during the process of collaborative problem-solving. In addition, on the Brief COPE, hybrid group participants showed a significant increase in acceptance and venting scores, suggesting that participants strengthened certain emotional regulation skills. Likewise, in qualitative interviews, participants reported benefits associated with identifying and expressing emotions, acceptance, cognitive reframing, and relaxation.

Conversely, those in the remote modality showed a significant decrease in social support, positive reappraisal, and venting coping methods from pre- to post-intervention. In qualitative interviews, participants in this modality attributed group-related benefits mainly to emotional regulation skills and social learning with some reports of problem-solving skill development. It is possible that remote groups adopted a more instructive style which privileged adoption of individual-level coping and problem-solving skills while impeding coping related to social support or sharing with others (i.e., venting). In sum, a particular understanding of the contextual facilitators of change and adapting interventions offers a promising venture for improving effectiveness in LMICs’ mental health outcomes [9, 21, 22].

Staff reported challenges in adapting the intervention to the remote modality (e.g., decreased participant involvement during the sessions, disconnections, privacy concerns), which impaired their ability to build a cohesive group dynamic effectively. Such constraints may have limited the development of critical elements such as confidence, trust, and emotional openness between the remote participants in the group [50, 51]. More work is needed to reassess and build understanding of how remote group participation may result in a decrease (rather than null effect) of social support and other coping mechanisms. Unpacking such potential to do harm is critical when considering scale-up of remote service delivery. Of note, the remote group included more men, younger participants, and those with less education than other groups, further complicating interpretability of results.

There were no significant changes in community efficacy and only trend effects in functional impairment from pre to post intervention in any modality. The lack of community efficacy effects may be explained by the dramatic impact of the COVID-19 pandemic on community practices, which, after the group ended, continued to impede ability to meet with or work together with other community members in meaningful ways in the longer term. Participants also reported Covid-related changes in daily life (i.e., losing employment, being forced to share more spaces with relatives, multi-tasking, restriction of community spaces and activities), which despite the intervention, remained highly challenging. These results coincide with findings from the cross-sectional studies in LMIC's in terms of the additive COVID-19 detrimental effect on the pre-existent vulnerabilities these communities struggle to face regarding MH and wellbeing [6].

### Limitations and next steps

As described earlier, this study is limited by the small sample size and the significant differences in sociodemographic variables (i.e., gender, age, and displacement) in the distribution across modalities, and the lack of a counterfactual/comparison group. In addition, the evaluator bias assessment resulted in three CPAs scoring an average of over 20 long strings at post. This lack of variability in responses might indicate bias introduced by the evaluator or a general lack of mental health problems. Further analyses to draw conclusions must be conducted. In addition, the telephonic component included as a mean to monitor and support participation was administered to all participants but had not a controlled assessment to evaluate its incidence. Further studies should assess the value of this resource's active incidence. Moreover, these results provide preliminary support for conducting additional work, and these shortcomings will be addressed in a subsequent RCT with a larger sample, which can account for potential confounding factors (e.g., pandemic-related changes). Based on the results of this pilot, the intervention protocol will be revised to strengthen guidelines for adapting procedures to remote modalities. Qualitative results also suggest the importance of including a standardized emotional regulation measure to investigate this reported facilitator of change.

This pilot revealed the potential benefit of a culturally-sensitive community-based psychosocial support intervention and the ability of the various modalities to meet the unique needs of different groups and individuals and, therefore to help to overcome implementation challenges, suggesting that multiple options of access should be made available and that participants should have an opportunity to select the modality that best suits them. For this reason, the RCT will include consistent adaptations to the findings (e.g., including an emotional regulation scale, changing the community support scale) and will offer remote and in-person options, available according to participant preferences (Additional file 1: Pilot Dataset).

## Conclusions

This pilot study provides a preliminary assessment of acceptability, feasibility and outcomes associated with a novel community-based intervention adapted to the COVID-19 context to serve conflict-affected adults in Quibdó, Colombia. Although additional work is needed to provide a more rigorous test of effectiveness, results suggest that this task-shifting approach, in which group sessions are facilitated by trained community members, is feasible and associated with improved mental health outcomes when implemented through in-person, remote, or hybrid modalities, with important caveats regarding risks associated with remote implementation. This study suggests that community-based models can and should be adapted for the COVID-19 pandemic and other contexts that require remote or hybrid service delivery, but that potential barriers and risks should be assessed and addressed, including reduced potential for benefits associated with social support and peer learning during remote implementation. This pilot study will inform implementation of a subsequent RCT, as well as scale-up of the model for use with other communities on the Pacific coast, such as indigenous and Venezuelan displaced adults. Indeed, this aligns and contributes to the Colombian peace accords and victims' restitution policies [52–54] framework that prioritizes adequate MHPSS provision as a restitution priority to the conflict-affected populations.

Together, these studies can support researchers in adapting and testing multi-modal interventions, provide guidance to practitioners, and help to inform public policies and programming designed to strengthen local and global responses for addressing MHPSS needs in LMICs during the COVID-19 pandemic.

### Supplementary Information

**Additional file 1**: Pilot Dataset.

## Data Availability

All data generated or analysed during this study are included in this published article [and its supplementary information files].

## Appendix

### Intervention sessions

**Table:**

Session #	Activity	Description
1	Introduction	Presentation of the goals and structure of the intervention to the participants
*Collaborative problem-solving sessions*		
3–5–7	Problem solving (PM+)	Address both psychological problems (e.g., stress, fear, feelings of helplessness) and, where possible, practical problems (e.g., livelihood problems, conflict in the family and so on) (WHO, 2018)
*Expressive sessions*		
2	Graphic Representation	Represent emotions, thoughts, and actions using pictorial means
4	Paper Mask	Recognize and represent the various forms of emotional manifestations through the design of a mask that represents the participant emotions
6	Dance, Expression and Corporal Movement	Express and reproduce through movement the inner world of the participants
8	Hero’s history	Recognize and express the emotions and thoughts that arise in relation to the finalization of the intervention

## Abbreviations

LMIC: Low-and middle-income countries
PTSD: Posttraumatic stress disorder
IDP: Internally displaced populations
MHPSS: Mental health and psychosocial support
WHO: World Health Organization
TSM: Task-Shifting Models
mhGAP: Mental Health Gap Action Program
PM+: Problem Management Plus
CB-PSS: Community-based psychosocial support
ACOPLE: Association of Organizations for Emotional Support
CPA: Community Psychosocial Agents
CETA: Common-Elements Treatment Approach
RCT: Randomized controlled trial
FGD: Focus group discussion
ANOVA: Mixed analyses of variance
RM-ANOVA: Mixed repeated measures analyses of variance
